# Effect of Thermal Drying and Chemical Treatments with Wastes on Microbiological Contamination Indicators in Sewage Sludge

**DOI:** 10.3390/microorganisms8030376

**Published:** 2020-03-07

**Authors:** Andreia F. Santos, Cátia P. Santos, Ana M. Matos, Olga Cardoso, Margarida J. Quina

**Affiliations:** 1 CIEPQPF-Centre of Chemical Processes Engineering and Forest Products, Department of Chemical Engineering, University of Coimbra, 3030-790 Coimbra, Portugal; catia4patricia@gmail.com (C.P.S.); guida@eq.uc.pt (M.J.Q.); 2 CIEPQPF-Centre of Chemical Processes Engineering and Forest Products, Faculty of Pharmacy, University of Coimbra, 3000-548 Coimbra, Portugal; anamatos@ci.uc.pt (A.M.M.); ocardoso@ci.uc.pt (O.C.)

**Keywords:** sewage sludge, organic wastes, pathogenic contamination, soil application, sanitation

## Abstract

This work aims to evaluate the microbiological contamination of sewage sludge (SS) collected in urban wastewater treatment plants (WWTP) from Portugal. Two types of SS were considered: urban mixed (UM) and from anaerobic digestion (AD). The two types of samples were characterized in relation to the main physical and chemical parameters, as well as the microbiological contamination (*Escherichia coli* and *Salmonella* spp). Then, sanitation tests were conducted through thermal drying and chemical treatments. Towards a circular economy, industrial alkaline wastes (green liquor dregs - GLD, lime mud, coal fly ash, eggshell) were tested as alternatives to lime. Only six out of nineteen samples complied with the legal limits for both microorganisms. However, drying at 130 °C sanitized selected samples below the *E. coli* limit, regardless of the initial moisture or contamination. Additionally, CaO (obtained from eggshell) led to the complete elimination of *E. coli* at any dosage studied (0.05–0.15 g/g SS_wet basis_). GLD evidenced the ability to reduce *E. coli* contamination at room temperature, but not enough to comply with the legal limit. In general, this work highlights the need to sanitize the SS before its application to the soil, and the positive role of some wastes on this goal.

## 1. Introduction

The circular economy framework encourages the recycling of organic wastes as a source of renewable energy and/or for increasing the organic matter in the soil after a stabilization process. For that purpose, sewage sludge (SS) produced in urban wastewater treatment plants (WWTP) represents a huge flow in developed countries that requires appropriate management. In fact, due to the global population growth, it is estimated that more than 13 Mt of SS on a dry basis will be generated by the European Union (EU) countries in 2020 [1,2]. The options for its proper management have been agriculture reuse, composting followed by soil application, incineration, landfill, or others [3]. Over the years, the EU policies have recommended a drastic reduction in landfill disposal, while a significant increase in incineration with energy recovery has been observed in some countries (e.g., in Germany and Slovenia). Regarding the SS recycling, in 2015, agriculture applications (directly or after composting) represented the main final disposal, whereas the prevailing technology can be very distinct in each Member State [1,4].

Due to its high organic matter and essential nutrient (P and N) contents, the agronomic application of SS can improve not only soil properties but also crop yield [5,6]. Indeed, several physical and chemical benefits have been shown when SS is used as a soil conditioner [7]. For example, Latare et al. [8] showed a statistically significant positive impact on the rice–wheat system, suggesting control in the doses of SS used and regular monitoring of metals build up not only in soil but also in plant parts to avoid food chain contamination. Moreover, Shu et al. [9] highlight the gains in the soil phosphorus using alkaline treated SS. Mayer et al. [10] emphasize the value of P recovery, namely through the application of SS in soil. A recent Swedish government report recommends that at least 60% of the P present in SS should be recycled (WWTP > 20000 p.e), discouraging the use of SS for non-agricultural applications (e.g., landscaping), where P is not valorized. Additionally, the European Sustainable Phosphorus Platform supports the use of SS as fertilizer [11], emphasizing the reduction of fertilizer costs with a positive impact on agriculture. However, awareness of risks is fundamental, particularly regarding potentially toxic metals, pathogenic microorganisms, and organic contaminants (some of them persistent organic compounds, pharmaceutical, and cosmetic residues). Recently, the problem associated with microplastics in SS has also emerged [12].

In the EU, the use of SS on soil must comply with the limits established in European Directive 86/278/EEC, which regulates the concentration of potentially toxic metals in the SS and in the receptor soil [13]. This directive is not updated and important pollutants (e.g., pathogenic organisms) that pose a risk to human health and environment have not been included [1,14]. Therefore, most EU Member States have released legislation including additional pollutants and with stricter limits. The legislation in Portugal (Decreto Lei n.° 276/2009) limits not only the concentration of potentially toxic metals, but also the concentration of organic compounds (e.g., PAH, PCB, etc.), and the presence of microbiological indicators (*Escherichia Coli—E. coli*, and *Salmonella* spp.). More specifically, the *E. coli* enumeration should be less than 1000 CFU/g (3 log CFU/g) and the *Salmonella* spp. should be absent in 50 g of the fresh sample.

Commonly, to fulfill the sanitation requirements in SS, additional processes are necessary to those currently existing in a WWTP, namely biological (anaerobic digestion, composting), drying (thermal drying, bio drying, and solar drying), radiation (UV), thermal processes (pasteurization, incineration, pyrolysis, and gasification), and/or chemical addition (lime addition, etc.) [7]. One of the most common treatments is pasteurization, and the EU and US EPA guidelines are the exposure of SS at 70 °C for 30 min, while at 80 °C only 10 min may be required [15,16]. Indeed, Kjerstadius et al. [17] report a reduction in *E. coli* contamination from 6.01 to < 1 log CFU/g at 70 °C for 60 min. In addition, despite the initial sample revealed the presence of *Salmonella* spp., the same was not verified after thermal treatment. Astals et al. [18] confirmed that at 80 °C, 14 min is enough to reduce the *E. coli* contamination from 4.19 to 1 log CFU/g. Regarding the effect of chemical treatments, it has been reported that pH ≤ 4 or ≥ 10 are adverse for microorganism survival. In particular, Ca(OH)_2_ is generally applied as a chemical conditioner to promote an increase in pH. Gantzer et al. [19] and Østensvik et al. [20] showed that the sanitation with Ca(OH)_2_ allowed a reduction in *E. coli* contamination from 5.70 to < 1 log CFU/g and from 7.05 to 2.06 log CFU/g, respectively. There is a window of opportunity to use alkaline waste instead of reagents as a sanitizing agent. Indeed, industrial alkaline wastes produced in high quantities could be a valuable alternative, with a potential contribution to the circular economy. Some examples are: green liquor dregs and lime mud from paper and pulp mills, with pH > 10 [21]; industrial eggshell [22]; and coal fly ash from a coal thermoelectric power plants with pH near to 9 [18]. It is important to note that the composition of such waste must be controlled to avoid the introduction of harmful contaminates in the final product, namely potentially toxic metals and toxic and persistent organic compounds (e.g., polychlorinated dibenzo-p-dioxins and dibenzofurans). Lang et al. [23] performed a field experiment on the survival of *E. coli* in agricultural soil amended with SS. The results showed that the conventionally treated SS (by mesophilic anaerobic digestion or thermal drying) increased the *E. coli* contamination in soil. However, the survival of this microorganism was limited to three months, regardless of the timing of application or environmental conditions. In another study, Scaglia et al. [24], highlighted that the pathogenic contained in SS was strongly reduced during anaerobic processes. Thus, further studies are required to add clarity to this issue.

In this context, this study aims to assess the regulated pathogens of SS from Portuguese WWTP, through the quantification of *E. coli* and the evaluation of presence/absence of *Salmonella* spp. Additionally, sanitation processes such as thermal drying and chemical treatment with wastes were evaluated through *E. coli* enumeration. To the best of our knowledge, the present study addresses for the first time in this field the use of different types of industrial alkaline wastes as chemical conditioners, avoiding their landfill disposal and contributing to the circular economy.

## 2. Materials and Methods

### 2.1. Materials

Nineteen samples of SS from twelve different WWTP in mainland Portugal (Figure S1, Supplementary information) were collected to assess the variability of microbiological contamination: eight from activated sludge systems plants (UM1-UM8), and four from anaerobic digestion processes (AD1-AD4). In each WWTP, one sample was collected except for UM7 and UM8, where two samples were collected, and in AD1 where six samples were taken. AD1.2, AD1.3, and AD1.4 were collected in the same point (after centrifugation) for two months to assess the variability of contamination over time. AD1.2, AD1.5, and AD1.6 were selected to analyze the effect of the storage method on-site. Table 1 summarizes the characteristics of the samples and the conditions of storage in laboratory until analysis.

Four industrial wastes were selected to act as chemical conditioners during the sanitation tests. A composite sample of weathered coal fly ash (CFA) was collected from a landfill in a thermoelectric power plant. Green liquor dregs (GLD) and lime mud (LM) were obtained from a national pulp and paper mill. Eggshell sample (ES) was collected from an egg pasteurization industry. In addition, through the calcination of ES at 900 °C, it was possible to obtain calcium oxide (CaO). Calcium hydroxide (Ca(OH)_2_) from Sigma-Aldrich was used as a reference. Some properties of the additives used are present in Table S1 (Supplementary information).

### 2.2. Physical and Chemical Characterization

Moisture (H) and organic matter (OM) were determined based on EPA Method 1684. The pH and electrical conductivity (EC) were measured (Consort C1020) in an extract obtained at 1:10 (solid:liquid) ratio. Acid digestion with aqua-regia followed by quantification with flame atomic absorption spectroscopy (Analytik Jena ContrAA 300) was used to quantify the potentially toxic metals (Pb, Zn, Cu, Cr, Cd, and Ni) and the major elements (Ca, Na, K, and Mg). Nitrogen was measured by the Kjeldahl method using the DKL and UDK units from VELP Scientifica. Total phosphorus was determined by colorimetric analysis using a UV-Vis spectrophotometry (wavelength of 650 nm) following the EPA method 365.3. All the parameters were measured in duplicate for each sample.

### 2.3. Microbiological Characterization

The *E. coli* enumeration was determined based on ISO 16649-2:2001. The initial suspension was prepared with 10 g of SS and 90 mL of tryptone salt. Then, three decimal dilutions were done (10^−2^, 10^−3^, and 10^−4^) from the suspension. Both initial suspension and dilutions were inoculated in tryptone-bile-glucoronic (TBX) medium using the pour plate method and incubated at 44 °C for 18–24 h. All the tests were performed in duplicate. Plates containing 30–300 blue colonies (characteristics of *E. coli*) were considered for counting. 

The presence/absence of *Salmonella* spp. was evaluated based on ISO 6579:2002. The procedure was divided into four steps: pre-enrichment, selective enrichment, isolation, and biochemical confirmation. For pre-enrichment, 50 g of SS were inoculated in 450 mL of buffered peptone water and incubated at 37 °C for 24 h. For selective enrichment, 0.1 mL of that culture were transferred to 10 mL of Rappaport-Vassiladis (RSV) broth and incubated at 41.5 °C for 24 h, and 1.0 mL were transferred to 10 mL of Muller-Kauffmann tetrathionate/novobiocin (MKTTn) broth and incubated at 37 °C for 24 h. Each obtained culture was inoculated in Xylose Lisine Deoxycholate agar (XLD) and Kektoen media, incubated at 37 °C for 24 h, for colonies isolation. Isolated colonies were selected to obtain a pure culture in Tryptone Soy Agar (TSA) medium at 37 °C for 24 h, for posterior identification through API^®^20E system. All the tests were performed in duplicate.

### 2.4. Sanitation Studies

#### 2.4.1. Thermal Treatment

This work is part of a broader project (Dry2Value), which aims to develop an industrial dryer to be used in WWTP to help with subsequent SS management operations. Thus, the thermal treatment conditions were selected considering the operation conditions foreseen for the dryer. The SS samples (UM4, AD1.3, and AD1.4) were dried at 70, 100, and 130 °C until 50% and 30% moisture to evaluate the effect of the temperature on *E. coli* enumeration. Based on previous studies [25], about 40 g of SS were shaped in small cylinders (5 mm diameter and 30 mm length) and dried in a Moisture Analyzer, Precisa XM50. The weight was recorded until achieving the required moisture. Also, the temperature inside the samples was monitored with a Pico Technology thermocouple data logger. The dry samples were stored in sterile flasks and kept at 4 °C until analysis within a maximum period of 48 h.

#### 2.4.2. Chemical Treatment

Four alkaline industrial wastes (GLD, LM, ES, CFA), CaO (obtained from calcined eggshell) and Ca(OH)_2_ (selected as a reference) were tested in the sanitation of SS. Thus, in sterile flasks, the additives were mixed with the SS sample (UM4) in a specific dosage at room temperature. Table 2 shows the conditions used in the chemical treatment assays.

### 2.5. Statistical Analysis

The statistical analysis of data includes mean and standard deviation calculations and one-way ANOVA (*p* < 0.05). Moreover, pairwise comparisons are performed to identify the statistically significant differences through the Tukey HSD test (*p* < 0.05).

## 3. Results and Discussion

### 3.1. Physical and Chemical Properties of the Sewage Sludge

Table 3 summarizes the main physical and chemical properties of the SS samples grouped into urban mixed (UM) and anaerobic digested (AD). The standard deviation was inferior to 5% for all parameters. These properties determine the beneficial or detrimental effect on the land application.

Both UM and AD samples are characterized by a pH near to neutrality, and thus a helpful effect is expected as a buffering agent in acidic soils (Portuguese case), at least in a short period [6]. The EC varied between 0.55 and 4.12 mS/cm, meaning that SS will not introduce high salinity into the soil. The moisture content affects the method of soil application (injected into the soil if moisture is high or spread on the soil top layer if moisture is low), and the release of offensive odors. The OM is highly dependent on the treatment technologies in the WWTP. It is observed that (as expected), in general, the OM in UM samples would be greater than in AD samples since through anaerobic digestion a fraction of OM is converted into biogas. Concerning the macronutrients, the UM samples contain significant higher levels of nitrogen when compared to AD samples (*p* < 0.05). However, the phosphorus content in both sample types is quite variable, but with agronomic interest [6,26]. Nevertheless, it is necessary to evaluate its bioavailability when applied to the soil. The levels of other nutrients, such as Mg, Ca, Na, and K, are lower and do not differ significantly in both samples (*p* < 0.05), except for Ca. The calcium content is higher in AD samples and this element can have a relevant positive effect in soil condition [22]. Indeed, acidic soils tend to have lower Ca concentrations, which impairs normal plant growth.

The concentration of potentially toxic metals (PTM) is a matter of concern foreseen in the legislation to consider the application of SS on the soil. According to the results in Table 4, it is possible to conclude that all the sludges samples analyzed are in compliance with the limits of Decreto-Lei n.° 276/2009, except for AD4. Indeed, in the AD4 sample the concentration of Cr, Zn, and Ni exceeded the legal limits. This contamination is due to the fact that this WWTP receives an industrial effluent from a metalworking industry with high concentrations of these refractory elements to biological processes. Overall, the PTM concentration is not a hampering factor to valorize Portuguese SS on the soil, which is in agreement with other reports in the literature [25,27].

### 3.2. Microbiological Contamination Assessment

**Figure 1:**
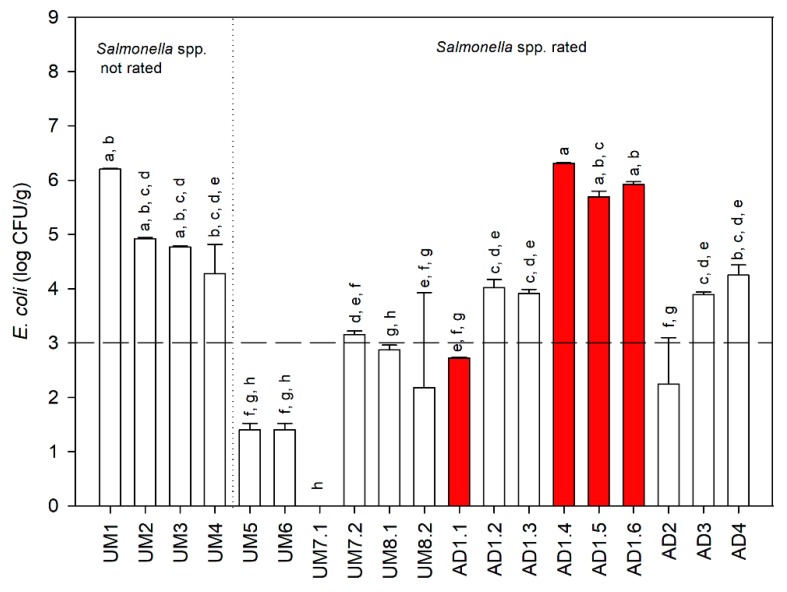
Enumeration of *E. coli* and absence/presence of *Salmonella* spp. (Results marked with the same letters are statistically similar (Tukey HSD test with *p* < 0.05); red bars correspond to the presence of *Salmonella* spp.).

Figure 1 summarizes the *E. coli* and *Salmonella* spp. contamination of the collected samples. The results indicate that 50% of the UM samples are below the legal limits established for both microorganisms, while only one AD sample complies with these values. Additionally, four out of fifteen samples revealed the presence of *Salmonella* spp. All AD samples came from mesophilic anaerobic digestion, which reveals the inability of this treatment to remove this microorganism [17,23]. Concerning the dewatering mechanism, the samples dehydrated in drying beds revealed in most of the cases a contamination below the legal limits for both microbiological indicators (even if the initial contamination was unknown). However, for a WWTP producing a large amount of SS, drying beds could not be a viable technology due to the space required [28]. In contrast, the dehydration through centrifugation or filtration did not allowed the removal of pathogens, as expected [29].

As said before, the samples AD1.2, AD1.3, and AD1.4 were collected at the same point in the WWTP (after centrifugation) for two months to evaluate the variability of contamination over time. The *E. coli* contamination in AD1.2 and AD1.3 was lower and statistically different from the AD1.4 (*p* < 0.05). Additionally, the sample AD1.4 revealed the presence of *Salmonella* spp. Furthermore, in the same WWTP, the samples AD1.2, AD1.5, and AD1.6 were selected to analyze the effect of the storage period until analysis. The AD1.2 sample was collected directly from the centrifuge, while AD1.5 and AD1.6 were stored in a heap in the WWTP one day and one week, respectively, after centrifugation. The results indicate that the storage in a heap was not appropriate for achieving concentrations lower than the sanitation requirements. Indeed, that procedure can even increase the contamination. According to Gantzer et al. [19], SS stored in heaps for six months without treatment showed a small reduction in *E. coli* contamination (about 1.5 log CFU/g).

**Figure 2:**
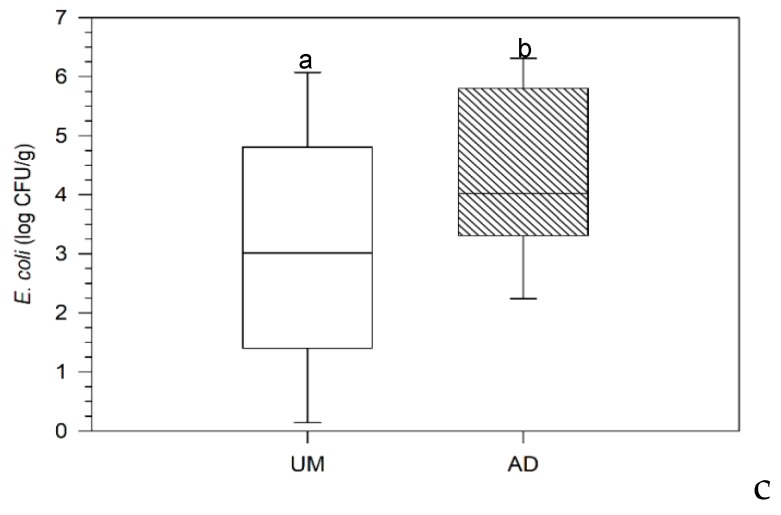
Variability of *E. coli* enumeration on UM and AD samples (Results marked with the same letters are statistically similar (Tukey HSD test with *p* < 0.05)).

Based on this assessment, the UM samples revealed higher variability on *E. coli* contamination than AD samples, (Figure 2), while the median value is lower in the former samples. In short, 68% of the samples analyzed could not be valorized in agriculture without any additional sanitation treatment. Thus, it is recommended to evaluate the possible ways to remove pathogens from SS to promote its application in the soil without compromising public health.

### 3.3. Sanitation Effect of Thermal Treatment

**Figure 3:**
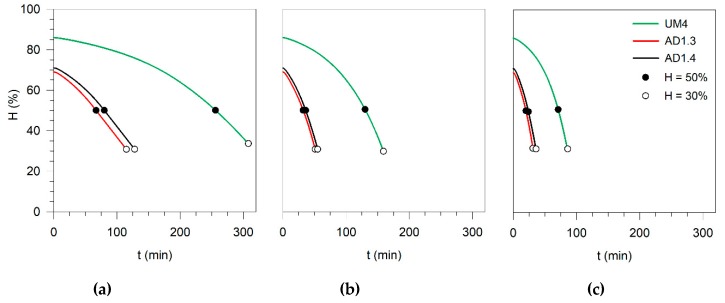
Moisture profile of UM4, AD1.3, and AD1.4 samples at: **(a**) 70 °C, (**b**) 100 °C, and (**c**) 130 °C.

As mentioned above, from the perspective of designing an industrial dryer, thermal treatments were conducted to evaluate the effect of temperature on reducing *E.* *coli* contamination. Figure 3a–c shows the moisture profile during the drying process for the three samples considered in this analysis (UM4, AD1.3, and AD1.4). The selection of these samples was based on the following criteria: (i) quantity of sample available for the tests; (ii) UM4 and AD1.3 with similar contamination, which allow us to compare the sanitation efficiencies of two different types of sludge; and (iii) AD1.4 has a high level of contamination, which enables to evaluate the efficiency of the thermal treatment in extreme cases.

**Figure 4:**
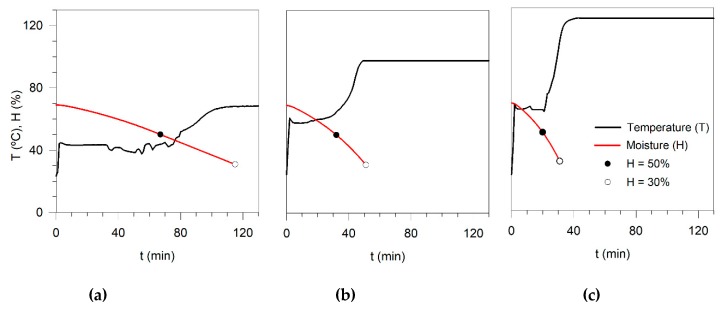
Temperature profiles inside the AD1.3 sample at a drying temperature of: (**a**) 70, (**b**) 100, and (**c**) 130 °C.

The moisture profiles of AD1.3 and AD1.4 were similar since the initial moistures are almost the same, 69% and 71%, respectively. In contrast, the UM4 sample required more time to dry until the required final moisture (50% or 30%). As expected, an increase in the drying temperature led to faster moisture reduction. However, during the drying of SS pellets, the temperature inside the solid is not constant and not equal to the chamber temperature, which can be a deterministic factor in pathogenic removal. Figure 4a–c presents the temperature profile inside the AD1.3 sample (as an example) during the drying at 70, 100, and 130 °C in the chamber.

As shown in Figure 4, a preheating stage occurs during about 5 min for all drying temperatures. Subsequently, the temperature becomes constant and equal to the wet-bulb temperature. In the end, the temperature inside the sample rises until it reaches the chamber temperature [25,30]. According to the temperature profile in Figure 4a, it is possible to notice that at 70 °C, the temperature inside the solid was about 44 °C until moisture reached 50% and then rose to 65 °C when 30% of moisture had been reached. At 100 °C, the SS temperature was 55 °C until it reached 50% moisture, while at 30% moisture was 85 °C. The drying at 130 °C allowed obtaining temperatures inside the solid of 65 and 115 °C at 50 and 30% moisture. The UM4 sample had longer drying times in the different drying periods. However, the temperature inside the solid was similar to the profiles of AD1.3 and AD1.4 (Figures S2 and S3, Supplementary information). Since *E. coli* withstands temperatures between 4 and 48 °C, it is important to consider the information discussed above. In some cases, the drying temperature could enhance the growth instead of the elimination of this microorganism. The temperatures and exposition times recommended by US EPA (2003) already consider the differences between the drying and the solid temperatures.

**Figure 5:**
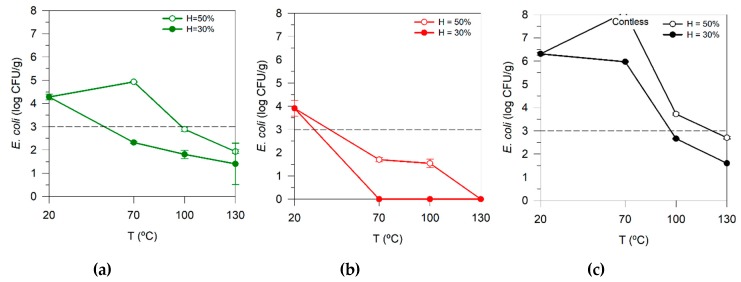
*E. coli* enumeration during the thermal treatment at different temperatures and moisture for (**a**) UM4, (**b**) AD1.3, and (**c**) AD1.4 samples.

Figure 5a–c shows contamination by *E. coli* in three samples (UM4, AD1.3, and AD1.4) before and after drying at 70, 100 and 130 °C. The thermal drying was performed until samples reach a moisture of 50% and 30%.

At 70 °C in the drying chamber, the moisture removal until 50%, the *E. coli* contamination in UM4 and AD1.4 samples were increased. As discussed above, in these conditions, the temperature inside the solids is about 44 °C, which is the recommended temperature for the incubation of this microorganism (ISO 16649-2:2001). However, the AD1.3 sample did not follow the same tendency despite the initial contamination being similar to the UM4 case. When samples achieve 30% moisture with a real temperature of 65 °C, UM4 and AD1.3 reach *E. coli* levels below the legal limit (< 3 log CFU/g). Similarly, Astals et al. [18] obtained a 3.19 log CFU/g reduction after drying at 65 °C for 35 min. Additionally, according to Strauch [31], at the same temperature and with a pure culture, it is possible to achieve a complete reduction within 30 min. The AD1.4 was the most problematic sample due to its high initial contamination level (6.31 log CFU/g). Indeed, only after drying at 100 °C until 30% of moisture was possible to reduce the *E. coli* below the legal limit. At 130 °C, regardless of the moisture or initial contamination, the drying allowed to decrease the *E. coli* to levels that enable their valorization in agriculture without compromising the public health.

### 3.4. Sanitation Effect of Chemical Treatment

**Figure 6:**
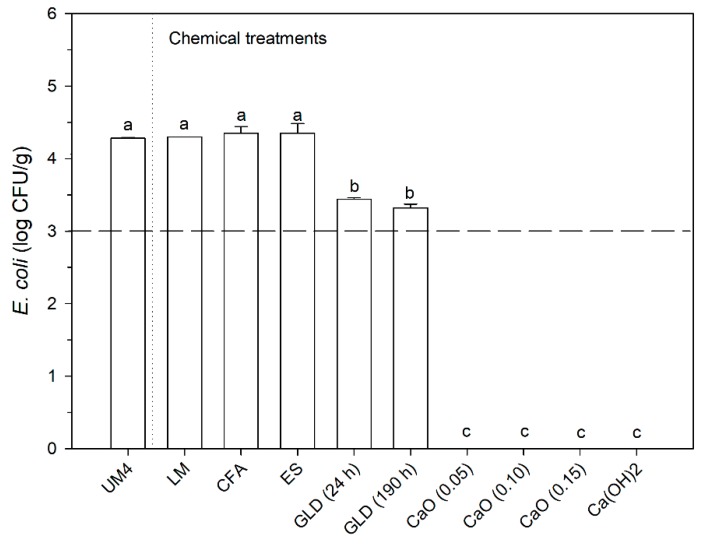
*E. coli* enumeration after chemical treatment of sample UM4 (Results marked with the same letters are statistically similar (Tukey HSD test with *p* < 0.05)).

The use of calcium oxide or hydroxide is very usual to raise the SS pH, and, consequently, eliminate pathogens [29,32]. However, nowadays, the circular economy agenda opens a window of opportunities for using waste for environmental protection. In this context, industrial alkaline wastes were selected to act as a chemical conditioner and explore a way to avoid their disposal in a landfill. Despite their original alkaline pH (Table S1, Supplementary information), it is important to evaluate the final pH in mixtures at a specific dose (0.15 g additive/g SS_wet basis_). Table 5 shows the results of pH obtained in UM4 used as an example, and Figure 6 depicts the *E. coli* enumeration after the chemical treatment.

Through Table 5 and Figure 6, it is possible to conclude that LM, CFA, and ES are not suitable to eliminate *E. coli* in the dose tested since the pH in the mixture is not adverse for microorganism survival (4 ≤ pH ≤ 10). In contrast, the use of CaO (obtained from ES) and Ca(OH)_2_ (reference) allowed the complete elimination of *E. coli* (at least, it was not detected). Different dosages of CaO were tested to evaluate the removal efficiency using smaller quantities, and the results were the same. Indeed, the production of CaO and its application to sanitize SS can be an interesting alternative to manage the high quantities of ES. Also, it is possible to use lower doses (SS plus CaO), which decrease the costs of producing CaO from ES. In the case of GLD, the results showed a decrease of 0.83 log CFU/g in the initial contamination after 24 h. Since the legal limit was almost achieved, the sample was analyzed again after 190 h. However, the contact time did not significantly improve the reduction of the contamination levels. Probably, the initial contamination of the sample requires extreme pH conditions for a more significant reduction of *E. coli*. Moreover, the dosage of GLD must be optimized. Thus, in-depth studies should be conducted for this waste. Nevertheless, further studies are ongoing to use of GLD combined with the drying process to facilitate the sanitation of highly contaminated samples. In fact, a recent study also showed that GLD can have a positive effect on drying efficiency [33].

## 4. Conclusions

According to the physical and chemical characterization, the SS demonstrates the potential to be valorized in agricultural soil due to its organic matter and macronutrient load. Additionally, the PTM concentration is not a hampering factor to SS valorization since the legal limits are in general fulfilled. However, the same was not observed for microbiological contamination. Only 32% of the samples comply with the legal restrictions for both microorganisms analyzed. *Salmonella* spp. was detected in four out of fifteen samples. Additionally, the *E. coli* contamination was higher in AD samples when compared to UM samples (*p* < 0.05). These results indicate that anaerobic mesophilic digestion may be insufficient to sanitize the samples and may even promote an increase in microbiological contamination. Regarding the thermal treatment assays, the results suggest that only the drying at 130 °C allowed to reduce the *E. coli* contamination below the legal limits for both moistures (50% and 30%). It was possible to conclude that the operation conditions are dependent on the initial contamination and the temperature inside the solid. The chemical sanitation tests demonstrate the efficiency of CaO to the complete elimination of *E. coli*. Additionally, the GLD also showed a significant reduction after 24 h (0.83 log CFU/g) but not enough to comply with the legal limits. Overall, this work reminds the importance of create alternatives to sanitize the SS before its application on the soil.

## Figures and Tables

**Table 1:** Characteristics of the samples and conditions of the analysis.

Sample	Dehydration Mechanism	Δt (days)	T_storage_ (°C)	Sample	Dehydration Mechanism	Δt (days)	T_storage_ (°C)
UM1	Centrifugation	1	T_R_	AD1.1	No dehydration	0	T_R_
UM2	Centrifugation	1	T_R_	AD1.2	Centrifugation	0	T_R_
UM3	Centrifugation	1	T_R_	AD1.3	Centrifugation	1	4
UM4	Filtration	4	4	AD1.4	Centrifugation	0	T_R_
UM5	Drying bed ^a^	3	T_R_	AD1.5	Centrifugation	1	T_R_
UM6	Drying bed ^a^	3	T_R_	AD1.6	Centrifugation	7	T_R_
UM7.1	Drying bed ^a^	3	T_R_	AD2	Drying bed ^a^	3	T_R_
UM7.2	Drying bed ^b^	22	T_R_	AD3	Drying bed ^a^	3	T_R_
UM8.1	Drying bed ^a^	3	T_R_	AD4	Drying bed ^a^	3	T_R_
UM8.2	Drying bed ^b^	22	T_R_				

Δt—storage period in the laboratory (days); Tstorage—storage temperature; T_R_—room temperature (~20 °C); a—for about 6 months; b—for less than 1 month.

**Table 2:** Chemical sanitation conditions.

Additives	Dosage (g_additive_/g_SS_)	Contact Time (h)
LM	0.15	24
CFA	0.15	24
ES	0.15	24
GLD	0.15	24, 190
CaO	0.05, 0.10, 0.15	24
Ca(OH)_2_	0.15	24

**Table 3:** Properties of sewage sludge (SS) samples analyzed in this study.

Samples	Parameters									
	pH	EC (mS/cm)	H (%)	OM (%)	P_2_O_5_ (%)	N_Kjeldahl_ (%)	K_2_O (%)	CaO (%)	MgO (%)	Na_2_O (%)
UM1	7.07	4.12	86.8	75.3	0.99	7.58	0.79	0.09	0.51	0.09
UM2	7.11	2.44	83.1	79.7	0.87	9.21	0.30	0.18	0.40	0.07
UM3	7.15	2.86	84.7	80.2	2.59	8.34	0.35	0.12	0.41	0.11
UM4	6.53	2.17	86.3	67.1	2.15	5.68	0.42	0.54	0.89	0.19
UM5	6.05	1.44	59.8	61.0	0.62	4.80	0.17	0.19	0.13	0.02
UM6	6.87	1.71	84.6	59.0	2.89	4.81	0.15	1.40	0.42	0.05
UM7 *	7.08	3.15	90.4	60.8	1.86	5.38	0.35	1.18	0.88	0.18
UM8 *	7.21	2.38	83.5	76.2	0.44	7.21	0.27	1.04	0.36	0.23
AD1 *	6.71	1.72	71.0	63.7	3.83	3.89	0.22	5.50	0.38	nd
AD2	7.29	0.55	48.0	50.4	1.63	3.28	0.10	1.38	0.24	nd
AD3	6.96	2.74	79.1	64.5	0.60	4.60	0.31	2.81	0.47	0.24
AD4	7.18	1.85	82.0	53.6	0.76	3.59	0.11	4.87	0.27	0.03

Note: with the exception of moisture, percentages were calculated on dry basis; UM7 *—mean values obtained for UM7.1 and UM7.2; UM8 *—mean values obtained for UM8.1 and UM8.2; AD1 *—mean values obtained for AD1.1 to AD1.6; EC—electrical conductivity; H—moisture; OM—organic matter.

**Table 4:** Potentially toxic metals (PTM) concentration (mg/kg) in urban mixed (UM) and anaerobic digestion (AD) samples and legal limits according to Portuguese law.

PTM (mg/kg)	Legal Limits *	UM Samples				AD Samples			
		Range	Mean	Median	SD	Range	Mean	Median	SD
Pb	750	26.3–155.4	63.9	49.5	42.9	19.8–38.8	25.7	22.6	7.54
Cr	1000	18.7–292.6	97.3	70.8	94.4	20.0–120.1 ^a^	55.8	27.4	42.2
Zn	2500	267.5–1026.3	616.3	548.82	229.1	331.2–1093.3 ^a^	841.5	1019.8	343.2
Cd	20	nd				nd			
Cu	1000	157.4–331.0	241.5	232.8	66.7	297.8–440.9	390.3	379.8	57.5
Ni	300	18.1–215.2	99.5	97.47	72.9	20.9–62.9 ^a^	36.0	24.3	19.0

* According to Decreto-Lei n.° 276/2009; ^a^ These values do not include the AD4 sample because the PTM concentration was higher than for regular SS samples: Cr = 1676.8 mg/kg; Zn = 9157.1 mg/kg; Ni = 2748.6 mg/kg; nd—not detected; SD—standard deviation.

**Table 5:** Final pH of the SS after chemical treatment with additives.

Additives	Mixture pH (0.15 g/g SS_wet basis_)
LM	8
CFA	7
ES	8
GLD	10
CaO	12
Ca(OH)_2_	12

## Supplementary Materials

Supplementary materials can be found at https://www.mdpi.com/2076-2607/8/3/376/s1.
