# Supplementary material for: Effect of Thermal Drying and Chemical Treatments with Wastes on Microbiological Contamination Indicators in Sewage Sludge
Source: Microorganisms. 2020 Mar 7;8(3):376. doi: 10.3390/microorganisms8030376 (PMC7142961; doi:10.3390/microorganisms8030376)
Supplement: Supplementary file 1 [file microorganisms-08-00376-s001.pdf]

microorganisms

MDPI

Article

Effect of thermal drying and chemical treatments with wastes on the sanitation of sewage sludge

Andreia F. Santos [1,*], Cátia P. Santos [1], Ana M. Matos [2], Olga Cardoso [2] and Margarida J. Quina [1]

[1] CIEPQPF - Centre of Chemical Processes Engineering and Forest Products, Department of Chemical Engineering, University of Coimbra, Coimbra, Portugal
[2] CIEPQPF - Centre of Chemical Processes Engineering and Forest Products, Faculty of Pharmacy, University of Coimbra, Coimbra, Portugal
* Correspondence: affs@eq.uc.pt

Supplementary information

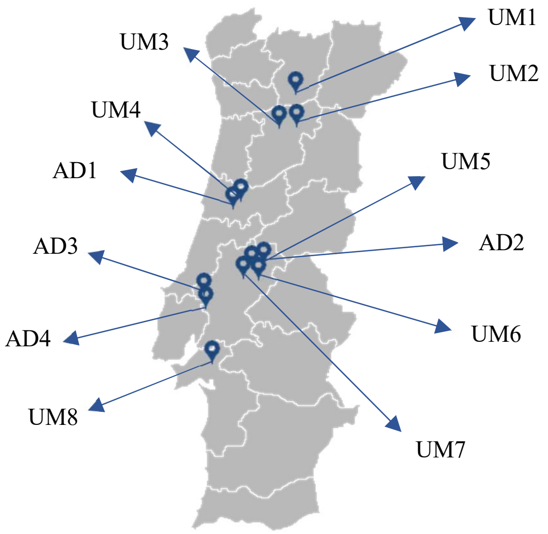

Figure S1. WWTP in mainland Portugal where SS were collected.

Table S1. Properties of the additives used.

Parameters *	Additives				
	CFA	GLD	ES	LM	CaO
pH	8.6	10.3	9.10	10.4	12.0
OM (%)	nd	nd	4.30	14.4	nd
Moisture (%)	0.07	20.8	1.10	nd	nd
EC (mS/cm)	0.49	16.6	0.21	1.20	nd
P_2O_5 (%)	0.2	0.15	0.29	nd	nd
K_2O (%)	2.4	0.06	0.10	nd	nd
MgO (%)	1.5	16.8	0.72	nd	nd
CaO (%)	1.4	52.9[(a)]	86.1[(a)]	nd	92.4

* the % was calculated on a dry basis, except for moisture; [(a)] these values are reported in $CaCO_3$; nd – not determined.

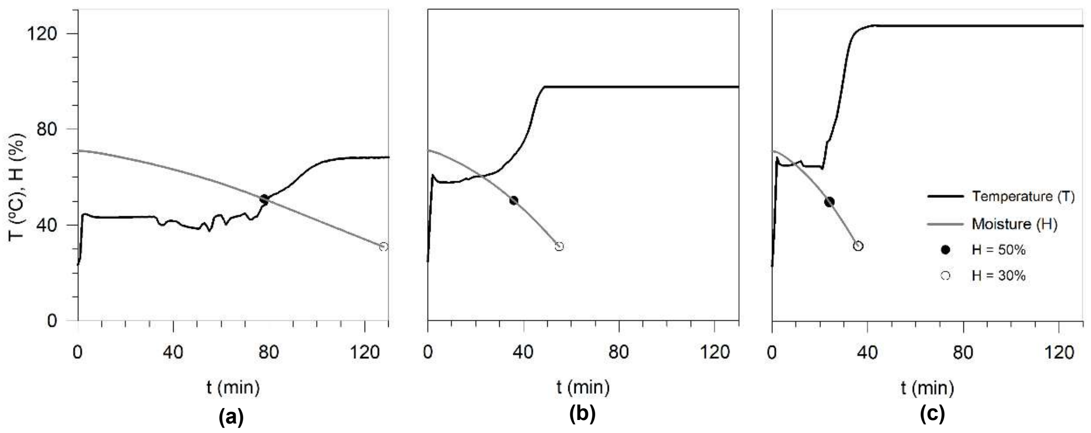

Figure S2. Temperature profiles inside the AD1.4 sample at a drying temperature of: (a) 70 °C, (b) 100 °C, and (c) 130 °C.

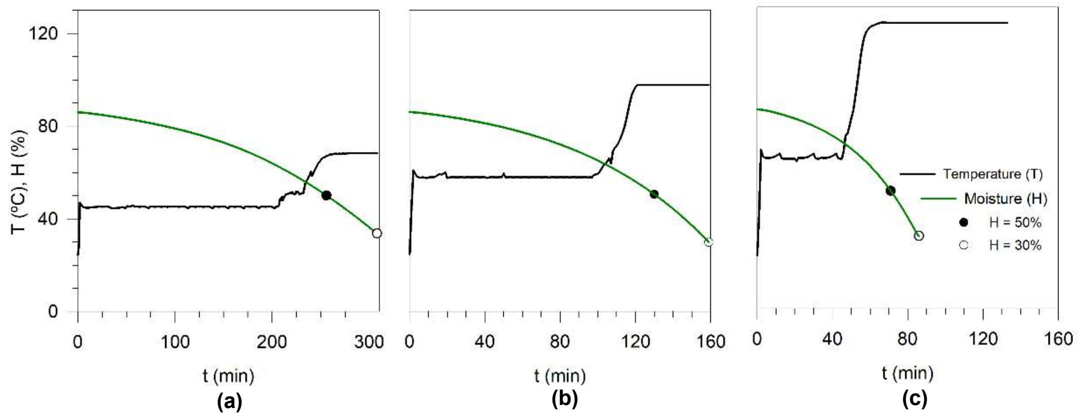

Figure S3. Temperature profiles inside the UM4 sample at a drying temperature of: (a) 70 °C, (b) 100 °C, and (c) 130 °C.
